# Editorial: Long Term Impact of War, Civil War and Persecution in Civilian Populations

**DOI:** 10.3389/fpsyt.2021.733493

**Published:** 2021-08-06

**Authors:** Thomas Wenzel, Meryam Schouler-Ocak, Thomas Stompe

**Affiliations:** ^1^World Psychiatric Scientific Section on Psychological Aspects of Torture and Persecution, Geneva, Switzerland; ^2^Psychiatric University Clinic of Charité, St. Hedwig Hospital, Berlin, Germany; ^3^Department of Psychiatry and Psychotherapy, Medical University of Vienna, Vienna, Austria

**Keywords:** war, torture, mental health, psychological trauma, transcultural aspects

This special volume of “Frontiers in Psychiatry” has a focus on different aspects of extreme violence, human rights violations, and mental health. The subject is unfortunately highly relevant, as in spite of a growing number of international conventions, statements and contracts, violence and violations of the human rights covered by this documents, is rather increasing than diminishing on a global scale. International humanitarian and human rights standards have defined actions as not permissible “under and circumstances, whatever” (1, 2). This reflects the concept of “natural law” or “jus cogens,” i.e., the understanding, that protection against some extreme acts of violence is obviously a basic concept of human ethics and that consequently protection must be given in an equal degree to everyone. The principle further reflects, that this acts have been documented not only to damage democracy, economy, and the development of peaceful civil society, but also to have a severe impact on public and especially on public mental health (3) to a degree that it might create more challenges then even the most commonly discussed Psychiatric illnesses, such as schizophrenia. Violence is destructive in two ways. On the one hand it destroys necessary health and other infrastructure and leads to brain drain of health care experts (4), while on the other hand it creates an increase in needs for that services through deprivation and physical and psychological injuries. Understanding long-term impact requires complex models especially in regard to the impact on populations, as explored by Musisi and Kinyanda in African communities. DroŽdek et al. have explored the limitations of the common models that are based on PTSD as major impact indicator and proposes more comprehensive approaches for a better understanding of trauma. Kienzler and Sapkota have explored the long – term impact in Nepal, a region that has experienced civil war and human rights violations for decades (5). Extreme acts such as torture, and especially sexual torture, civil war, or genocidal actions have been further shown to have an impact not only on the immediate victims, but also on family members and society. Hoffman and Shrira have explored this important issue that further underlines the need of prevention of violence. Recent data also show that there is a transgenerational impact (6), probably even by epigenetic mechanisms (7). Shahini et al. have documented the impact on (former civilian) war veterans together with their family members in Kosovo, a country facing ongoing social and economic problems (8). Increase of suicide and suicidal ideation is a frequently observed problem, and Marie Ingabire and Richters have explored this problem in Congolese refugees in Rwanda.

## Who are The Perpetrators?

There is a wide range of forms and perpetrators of such human rights violations, from obvious ones such as abductions and enslavement of Yezidi women by “non-state actors” such as ISIS/DAESH in Iraq and Syria (9, 10), torture and violence against women and other groups in Iran, or actions frequently described as genocidal against minorities as in Maynmar, or in the people's republic of China. This development is also supported by the abuse of the term of “terrorist” by governments against opposition parties, minorities, and human rights defenders to justify what cannot be justified (11). Understanding how perpetrators are created is important not to justify their actions, but rather to better understand the impact on victims and especially to prevent future violence by early interventions. Further, community and group processes should receive special attention. Vyshka and Çomo have explored this aspect in Albania, a country still haunted by the shadows of a bizarre dictatorship.

## What Can Be Done?

In acts of violence affecting large populations as in the Rwanda genocide or atrocities in South Africa and Uganda, new models aim at supporting surviving victims and their family members that often have to live together with perpetrators in the same regions by “transitional” justice models like traditional courts and “justice and reconciliation” committees (12). This is expected to help to recreate post-conflict countries in reconfirmation of civil society and rule of law, and give at the same time psychosocial support and reconfirmation to victims. The needs of victims independent from medical and psychiatric concepts and traditional treatment models or diagnostical categories (13) are often summarized by three key aspects. They include a confirmation of what has happened, as perpetrators frequently deny the reality of atrocities besides their responsibility in these acts. Further survivors and their family members need confirmation that the acts were wrong and cannot be justified, and a guarantee of “non-repetition,” which is linked to the two earlier aspects. Refugees might need more immediate care and reliable protection, at a minimum until the situation in the home country has significantly improved, which might require transitional justice approaches as outlined above.

Trilesnik et al. are presenting the first data on the important international “RefuKey” stepped treatment project for refugee populations. Ekblad presents a model focusing on the factor of health literacy and human rights in displaced populations, as missing this aspect can increase the risk for long-term sequels in these populations.

The present collection of articles in “Frontiers” brings together articles on different so far insufficiently covered aspects of these problems from different regions of the world. We hope to contribute to a broader discussion on the subject and the editors and “Frontiers in Psychiatry” invite you to enjoy the excellent research and considerations presented by our authors.

## Author Contributions

The above co-authors served as co-editors of the special volume, and have given input to the editorial and the development of the special issue. TW served as the main writer for the editorial. All authors contributed to the article and approved the submitted version.

## Conflict of Interest

The authors declare that the research was conducted in the absence of any commercial or financial relationships that could be construed as a potential conflict of interest.

## Publisher's Note

All claims expressed in this article are solely those of the authors and do not necessarily represent those of their affiliated organizations, or those of the publisher, the editors and the reviewers. Any product that may be evaluated in this article, or claim that may be made by its manufacturer, is not guaranteed or endorsed by the publisher.
